# Bioassay-Guided Separation of *Centipeda minima* Using Comprehensive Linear Gradient Centrifugal Partition Chromatography

**DOI:** 10.3390/molecules25133077

**Published:** 2020-07-06

**Authors:** Ji Hoon Kim, Eun Ju Jung, Yun Jung Lee, En Mei Gao, Ahmed Shah Syed, Chul Young Kim

**Affiliations:** 1 College of Pharmacy and Institute of Pharmaceutical Science and Technology, Hanyang University, Ansan, Gyeonggi-do 15588, Korea; gg890718@gmail.com (J.H.K.); jejs2@naver.com (E.J.J.); sopihya@naver.com (Y.J.L.); rhdmsal@hanyang.ac.kr (E.M.G.); 2 Department of Pharmacognosy, Faculty of Pharmacy, University of Sindh, Jamshoro 76088, Pakistan; shahahmed454@gmail.com

**Keywords:** centrifugal partition chromatography, comprehensive linear gradient elution, bioactivity-guided isolation, 3-methoxyquercetin, brevilin A, *Centipeda minima*

## Abstract

A comprehensive linear gradient solvent system for centrifugal partition chromatography (CPC) was developed for the bioassay-guided isolation of natural compounds. The gradient solvent system consisted of three different ternary biphasic solvents types: *n*-hexane–acetonitrile–water (10:2:8, *v*/*v*), ethyl acetate–acetonitrile–water (10:2:8, *v*/*v*), and water-saturated *n*-butanol–acetonitrile–water (10:2:8, *v*/*v*). The lower phase of the *n*-hexane–acetonitrile–water (10:2:8, *v*/*v*) was used as the stationary phase, while its upper phase, as well as ethyl acetate–acetonitrile–water (10:2:8), and water-saturated *n*-butanol–acetonitrile–water (10:2:8, *v*/*v*) were pumped to generate a linear gradient elution, increasing the mobile phase polarity. We used the gradient CPC to identify antioxidant response elements (AREs), inducing compounds from *Centipeda minima*, using an ARE-luciferase assay in HepG2 cells, which led to the purification of the active molecules 3-methoxyquercetin and brevilin A. The developed CPC solvent systems allow the separation and isolation of compounds with a wide polarity range, allowing active molecule identification in the complex crude extract of natural products.

## 1. Introduction

For centuries, medicinal plants have been valuable sources of functional ingredients, playing an important role in health maintenance, as well as disease prevention and treatment. Natural medicinal plant products provide unlimited opportunities for nutraceuticals, functional foods, natural health products, and new drug leads due to the considerable chemical diversity they make available [1,2,3]. In order to identify bioactive compounds from natural extracts, bioassay-guided fractionation is a common approach, performed through the following process: (i) solvent extraction from crude materials, (ii) fractionation of the liquid–liquid extraction and bioassay, (iii) fractionation using open column chromatography or prep-HPLC and bioassay screening of each fraction, (iv) active molecule isolation from the active fractions, and (v) structural elucidation of the isolated molecules and the evaluation of their bioactivity [4]. However, loss of activity or failure of the bioactive compound isolation from crude extracts during the bioactivity-guided isolation are common problems. Generally, the degradation or irreversible adsorption in a solid matrix during the purification process are the main reasons for these concerns. In addition, natural products are complex mixtures containing multiple components, and the conventional bioassay-guided fractionation strategy is time- and resource-intensive. Therefore, a more practical method for herbal medicine-derived active component discovery and purification would be required [5]. In order to solve these problems, countercurrent separation could provide an excellent alternative, such as centrifugal partition chromatography (CPC) or high-speed counter-current chromatography (HSCCC). CPC is a type of support-free liquid–liquid partition chromatography, with a low risk of sample denaturation, as well as total sample recovery, and large load capacity that has been widely used for the separation of constituents with bioactivity [6,7,8,9,10]. Despite the above-mentioned advantages, the CPC-mediated bioactivity-guided separation has several complementary points: (i) most solvent systems are limited to separating constituents with a narrow polarity range, (ii) optimizing a biphasic solvent system is a time-consuming process, as the partition coefficient of each component of the two layers should be compared in different ratios of the various solvents using HPLC or TLC. For the CPC-mediated activity-guided separation, a gradient biphasic solvent system that extends an extreme polarity range would be required without calculating the K values [11,12,13,14].

*Centipeda minima* is a traditional medicine used for treating cancer, sinusitis, swelling, and rhinitis. Phytochemical studies have isolated chlorogenic acid, cryptochlorogenic acid, caffeic acid, rutin, isochlorogenic acid B, kaempferol-3-O-rutinoside, isochlorogenic acid A, isochlorogenic acid C, 3-methoxyquercetin, brevilin A, arnicolide D, and arnicolide C from *C. minima* [15,16]. A previous study reported on the antioxidant effect of *C. minima* through the activation of the nuclear factor erythroid 2-related factor 2 signaling pathway from [17]. Therefore, *C. minima* was selected as a model sample to identify the antioxidant response element (ARE)-inducing compounds using a comprehensive linear-gradient CPC.

This study aimed at developing a linear gradient solvent system for CPC in order to fulfill compounds with a wide polarity range for the screening and separation of active constituents from herbal medicines. Based on the conventional liquid–liquid extraction approach, *n*-hexane, ethyl acetate, and water-saturated *n*-butanol were chosen as less polar solvents in ternary biphasic solvent systems. Considering the settling time and upper/lower volume ratios, acetonitrile was used as the polarity modifier. The developed gradient solvent system was applied for the activity-guided purification with an ARE-luciferase reporter assay in HepG2 cells transfected with the *C. minima* ARE gene.

## 2. Results and Discussion

### 2.1. Gradient Biphasic Solvent System Selection

**Figure 1:**
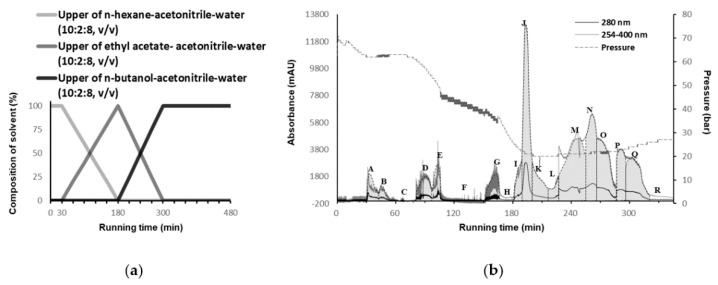
(**a**) Centrifugal partition chromatography (CPC) operation and chromatogram. Mobile phase composition with linear-gradient elution and (**b**) CPC separation chromatogram at 280 nm and scan mode: 254–320 nm. The details are described in Section 3.6. CPC procedure.

As described previously, *C. minima* extract (CME) consists of mixtures with a wide range of polarities that are well-represented by the various features of natural products (Figure S3). In order to separate these CMEs through bioassay-guiding, we developed a comprehensive linear gradient solvent system that covered a wide polarity range, from nonpolar to extremely polar, with no K value calculation necessity. As shown in Figure 1, the CME consisted of compounds with a wide polarity range, a comprehensive gradient CPC operation would thus be needed to recover all the introduced components. Since the gradient solvent systems should satisfy various polar compounds and stable stationary phases, the aqueous phase was used as the stationary phase and the organic phase was used as the mobile phase with increasing polarities. Then, the organic layer polarity was sequentially increased with *n*-hexane, ethyl acetate, and *n*-butanol. In addition, as *n*-butanol was less polar to achieve an appropriate partition coefficient for a very polar constituent, a ternary solvent system was introduced in order to separate a suitable polarity for very polar constituents [18,19].

As a result, gradient ternary biphasic solvent systems were divided into three polarity groups: nonpolar, intermediate, and polar conditions, consisting of *n*-hexane–X–water, ethyl acetate–X–water, and water-saturated *n*-butanol–X–water, respectively. X stands for the tested modifier solvents, which were methanol, ethanol, acetonitrile, or isopropanol in this study. The ternary biphasic solvent systems, phase ratio, and settling time are described in Table 1. According to the settling time and biphasic solvent ratio (Figure S1, Tables S1 and S2), acetonitrile was determined as a modifier solvent. Finally, a volume ratio of 10:2:8 of *n*-hexane–acetonitrile–water, ethyl acetate–acetonitrile–water, and water-saturated *n*-butanol–acetonitrile–water were selected as linear-gradient solvent systems. Thus, three ternary biphasic solvent systems were separately prepared, and the lower layer of *n*-hexane–acetonitrile–water (10:2:8, *v*/*v*/*v*) was used as the stationary phase. Then, the upper layer of *n*-hexane–acetonitrile–water, ethyl acetate–acetonitrile–water, and water-saturated *n*-butanol–acetonitrile–water (10:2:8, *v*/*v*/*v*) were pumped to generate a linear gradient elution, thereby increasing the mobile phase polarity to recover all CME compounds. In the case of isopropanol as a modifier solvent, the initial pressure was too high for the upper mobile phase to flow out in the actual CPC procedure.

### 2.2. Separation of the CPC Gradient Elution and ARE Induction Activity

CPC was operated in a linear gradient of three ternary solvent systems, as shown in Figure 1a. The upper phase of the three ternary solvent systems was introduced sequentially as the mobile phase. The mobile phase consisted of *n*-hexane–acetonitrile–water (10:2:8, *v*/*v*/*v*), ethyl acetate–acetonitrile–water (10:2:8, *v*/*v*/*v*), and water-saturated *n*-butanol–acetonitrile–water (10:2:8).

As shown in Figure 1b, eighteen high-resolution fractions (**A**–**R**) were obtained, and the overall CPC operating time was 360 min. The initial operating pressure was approximately 70 bar, then it began to decline after 80 min, and was then maintained at 25 bar for approximately 180 min.

According to the CPC chromatogram, eighteen fractions were collected and evaporated to dryness: **A** (30–42.5 min, 173.7 mg), **B** (42.5–55 min, 44.8 mg), **C** (55–80 min, 48.6 mg), **D** (80–97.5 min, 76.7 mg), **E** (97.5–110 min, 119.2 mg), **F** (110–150 min, 51.5 mg), **G** (150–167 min, 65.4 mg), **H** (167–180.5 min, 25.7 mg), **I** (180.5–189 min, 55.6 mg), **J** (189–200.5 min, 100.8 mg), **K** (200.5–217.5 min, 106.7 mg), **L** (217.5–227 min, 75.8 mg), **M** (227–255.5 min, 243.9 mg), **N** (255.5–267 min, 116.6 mg), **O** (267–285.5 min, 97.2 mg), **P** (285.5–295.5 min, 33.8 mg), **Q** (295.5–320.5 min, 82.4 mg), and **R** (320.5–residue in rotor, 2387.5 mg). After the CPC operation, the total sum of each fraction was 3905.9 mg when 4.0 g of crude sample was loaded. The recovery rate was 97.6% (Table 2).

**Figure 2:**
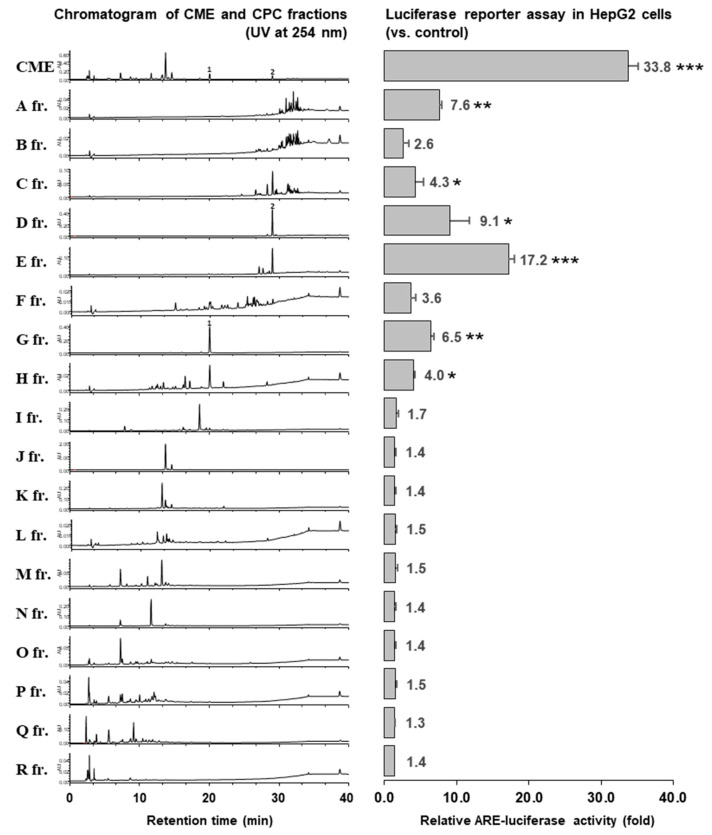
HPLC chromatograms and the relative antioxidant response element (ARE)-luciferase activities of the CPC-fractions (**A**–**R**). Each CPC fraction was analyzed using HPLC and the ARE induction activities were evaluated in ARE-HepG2 cells at concentrations applied at each assigned weight ratio (based on 30 μg/mL *C. minima* extract (CME)). Data are presented as the mean ±S.E. (*n* = 3). * *p* < 0.05; ** *p* < 0.01; *** *p* < 0.005 (compared with the vehicle-treated control).

**Figure 3:**
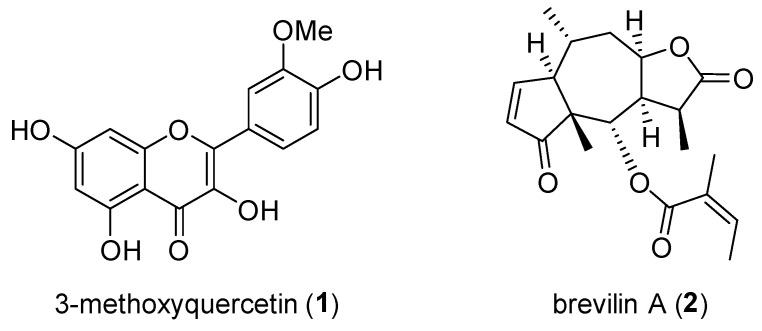
Purified compound identification. The chemical structures of compounds **1** and **2**.

The fractions were profiled using HPLC/UV at 254 nm, and we identified major peaks associated with the CME (fractions **D**, **G**, **J**, **K**, **M**, and **N**) as showed in Figure 2. We next evaluated the CME for the ARE-inducing activity at a concentration of 30 μg/mL, and 18 fractions were evaluated at the concentration applied at each assigned weight ratio (Table 2). The results showed that fraction **E** exerted the highest activity, followed by fractions **D**, **A**, and **G**. However, the activity of fraction **A** is not representative due to the following reasons: (i) it was treated at relatively high concentrations, (ii) the fraction chromatogram profiling shows the peaks of several substances, and (iii) compared with fraction **B**, the chromatogram profiling showed a similar activity that was proportional with the concentration (treated fractions **A** and **B** with concentrations of 1.33 and 0.34, **A** and **B** activities = 7.6- and 2.6-fold, respectively). Outside of that, fraction **I**–**R** are non-active. Overall, the fractions including compound **1** and **2** in the HPLC chromatograms of fraction **A**–**R** were evaluated to be ARE-inducing activity. Therefore, fractions **D**–**E** and **G** were further purified using preparative HPLC in order to obtain pure 3-methoxyquercetin (**1**) and brevilin A (**2**), respectively. The chemical structures of these compounds are shown in Figure 3. The physicochemical information of these compounds is indicated in Section structural information of the supplementary.

**Figure 4:**
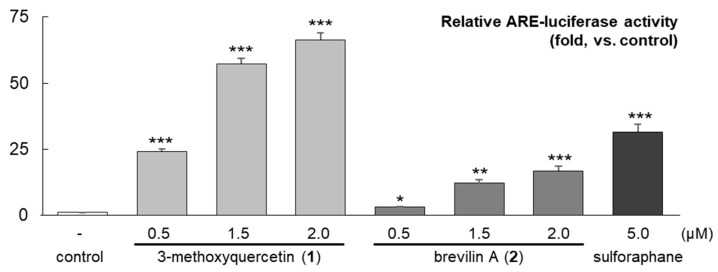
Relative ARE-luciferase activities of the purified compound. The ARE induction activities of compounds **1** and **2** were evaluated in ARE-HepG2 cells at concentrations of 0.5, 1.5, and 2.0 μM. Data are presented as the mean ±S.E. (*n* = 3). * *p* < 0.05; ** *p* < 0.01; *** *p* < 0.005 (compared with the vehicle-treated control).

The ARE induction activity of the isolated compounds from active fractions **D**–**E** and **G** was assessed using a luciferase assay in HepG2 cells at serial concentrations of 0.5, 1.5, and 2.0 μM. The results showed that 3-methoxyquercetin (**1**) and brevilin A (**2**) enhanced the ARE activity in a dose-dependent manner (Figure 4). The 3-methoxyquercetin (**1**)-derived ARE-inducing activity was prominent. Significant antioxidant and hepatoprotective activities have already been reportedly attributed to 3-methoxyquercetin indicated by its ability to prevent liver cell death during CCl_4_ intoxication [20].

## 3. Materials and Methods

### 3.1. Reagents and Materials

All solvents used for the CPC were of analytical grade and purchased from Daejung Chemical (Gyeonggi-do, Republic of Korea). The HPLC-grade solvents were obtained from Fisher Scientific (Pittsburgh, PA, USA). The *C. minima* sample was purchased from the Kyungdong Oriental Herbal Market, Seoul, the Republic of Korea, in May 2017. A voucher specimen (HYUP-CM-001) was deposited in the Herbarium of the College of Pharmacy, Hanyang University.

### 3.2. C. minima Extract Preparation

The dried *C. minima* was chopped to the appropriate size, extracted by reflux with methanol (three times for 2 h each), and the extract solution was absorbed using Diaion HP-20 resin for chlorophyll removal followed by lyophilization in order to obtain a 4.31%-yield dried extract (CME). The CME was stored at −20 °C until further use. For the cell treatment, the CME was dissolved in DMSO.

### 3.3. Ternary Biphasic Solvent System Preparation

In order to find a gradient elution solvent system capable of separating compounds with a wide polarity range, common solvents used for liquid–liquid extraction (*n*-hexane, ethyl acetate, water-saturated *n*-butanol, and distilled water) were fixed, and the polarity modifier solvents (methanol, ethanol, acetonitrile, and isopropanol) were tested. The various ternary biphasic solvent systems are listed in Table 1.

### 3.4. Settling Time and Phase Ratio Measurement

The settling time, which highly correlated with the retention of the stationary phase, was expressed as the time to form a clear layer between the two phases (1:1, *v*/*v*) when mixed. The phase ratio was calculated as the ratio of each phase after mixing the upper phase with the lower phase.

### 3.5. HPLC Analysis

CME and CPC peak fractions were analyzed using a Waters Alliance 2695 HPLC system coupled with a Waters 2996 PDA detector (Waters, Milford, MA, USA) with a Capcellpak UG120 C18 column (4.6 × 250 mm, 5 μm, Shiseido, Tokyo, Japan). Acetonitrile (0.1% formic acid, solvent A) and water (0.1% formic acid, solvent B) were eluted in a gradient mode: 0–20 min, 10–40% A; 20–25 min, 40–55% A; 25–30 min, 55–95% A; and 40 min, 95% A. The flow rate was 1 mL/min, and the injection volume was 10 μL. The diode array detector measured the UV spectrum over a range of 200–450 nm, and the chromatogram of the effluents was recorded at 254 nm. The system was controlled using Waters Empower™ 2 Chromatography Software.

### 3.6. CPC Procedure

An SCPC-1000 (Armen instrument, St-Ave, France) apparatus and a Spot Prep II HPLC instrument were combined to form a CPC system. The preparation of the biphasic solvent system for the linear gradient elution mode was designed as follows: the upper and lower phase solvents were prepared when the solvents were perfectly mixed and completely equilibrated. The 1000 mL volume of the CPC rotor was filled with a lower layer of *n*-hexane–acetonitrile–water (10:2:8, *v*/*v*/*v*) as the stationary phase at 50 mL/min in ascending mode at a speed of 500 rpm. Then, the rotation speed of the rotor was accelerated to 800 rpm, and the upper layer as the mobile phase was carried into the rotor in descending mode at a flow rate of 10 mL/min. The CPC rotor reached equilibrium at the immiscible biphasic state (320 mL of the stationary phase out of 1000 mL rotor volume, 69 bar). The elution was carried out in gradient mode with a mixed mobile phase of A (upper layer of *n*-hexane–acetonitrile–water, 10:2:8, *v*/*v*/*v*), B (upper layer of ethyl acetate–acetonitrile–water, 10:2:8, *v*/*v*/*v*), and C (upper layer of water-saturated *n*-butanol–acetonitrile–water, 10:2:8, *v*/*v*/*v*) with a flow rate of 10 mL/min for 0–30 min (100% A), 30–180 min (100% A–100% B), 180–300 min (100% B–100% C), and 300–480 min (100% C). Thereafter, methanol was pumped for washing at 50 mL/min, and all the remaining samples were recovered. The CME (4.0 g) was dissolved in 20 mL of mixed upper and lower phases and subjected to the CPC system. The system was controlled using Armen Glider CPC V5.0b.09 Software.

### 3.7. Cell Culture and Viability Assay

The construction of the HepG2-ARE cells (transfected Pgl4.37 [luc2P/ARE/ Hygro] (Promega)) was performed as described previously [21]. The HepG2-ARE cells were cultured in DMEM high glucose media (Hyclone, Logan, UT, USA) supplemented with 10% FBS (Hyclone, Logan, UT), 1% penicillin-streptomycin (Hyclone, Logan, UT, USA), and 1% hygromycin B (Invitrogen, Carlsbad, CA, USA). The cell viability was examined using a 3-(4,5-dimethylthiazol-2-yl)-2,5-diphenyl tetrazolium bromide (MTT) assay. HepG2 cells stably transfected with pGL4.37 (HepG2-ARE cells) were seeded at a density of 1 × 10^5^ cells/well in 24-well plates for 24 h. After serum starvation for 12 h and upon reaching an approximately 80% confluency, the cells were incubated with the major compounds for 24 h. The cells were treated with 50 μL MTT for 1 h. The formazan precipitate was dissolved in 1 mL of dimethyl sulfoxide (DMSO), and the absorbance was measured at 570 nm using a microplate reader.

### 3.8. ARE-Inducing Activity Assay

HepG2-ARE cells were seeded at a density of 1 × 10^5^ cells/well in 24-well plates for 24 h. The cells were starved for 12 h when they grew to approximately 80% of confluency and exposed to crude extract, CPC fractions, and purified compounds for an additional 24. Then, the cells were lysed using 120 μL of passive lysis buffer (Promega, Madison, WI, USA) in an ice rack and transferred into 1.5-mL tubes. The tubes were centrifuged at 1000 rpm for 3 min. Each supernatant (30 μL) in the centrifuged tube was reacted with 60 μL of assay substrate in a white 96-well plate. Finally, the luminescence was measured using an EnSpire multimode plate reader (PerkinElmer, Waltham, MA, USA). DMSO (below 0.1%) was used as a vehicle, which was the negative control. Sulforaphane was used as a positive control.

### 3.9. Protein Assay

The protein was determined using the Pierce Micro BCA protein assay kit with BSA as a standard (Pierce No. 23227; Thermo Fisher Scientific, Illinois, USA). Each standard and unknown sample lysate (10 µL) was replicated in a 96-well microplate. The working reagent (200 µL) was added to each well. The BCA reagents A and B were mixed in a 49:1 volume ratio. After 30 min of incubation at 37 °C, the plate was cooled at room temperature and the absorbance was measured at 562 nm using a microplate reader. The calculated value of the protein assay was used as a factor to correct for ARE-inducing activity.

### 3.10. Statistical Analysis

All data are presented as the mean ± standard error (S.E.) and the difference between the control and treatment groups was assessed using the Student’s t-test. * *p* < 0.05, ** *p* < 0.01, *** *p* < 0.005 were considered statistically significant.

## 4. Conclusions

In this study, a comprehensive CPC gradient solvent system was developed for bioassay-guided isolation from the CME. The upper phase of three ternary solvent systems with *n*-hexane–acetonitrile–water, ethyl acetate–acetonitrile–water, and water-saturated *n*-butanol–acetonitrile–water (each 10:2:8, *v*/*v*/*v*) eluted in a linear gradient mode, and the ARE-inducing molecule 3-methoxyquercetin (**1**) and brevilin A (**2**) were purified from the CME. The overall results of this study demonstrate that a linear gradient CPC with three ternary solvent systems is suitable for screening bioactive molecules from natural products, even when the compounds have a wide polarity range.

## Figures and Tables

**Table 1:** Phase ratios and settling times of each ternary biphasic solvent system.

Less Polar Solvent Systems				Medium Polar Solvent Systems				Polar Solvent Systems			
Solvent System	Volume Ratios (*v/v/v*)	Phase Ratios	Settling Time	Solvent System	Volume Ratios (*v/v/v*)	Phase Ratios	Settling Time	Solvent System	Volume Ratios (*v*/*v*/*v*)	Phase Ratios	Settling Time
H/M/W	10:1:9	50/50	12 s	EA/M/W	10:1:9	58/42	19 s	B/M/W	10:1:9	50/50	26 s
	10:2:8	50/50	12 s		10:2:8	58/42	20 s		10:2:8	56/44	3 m 17 s
H/E/W	10:1:9	50/50	>4 m	EA/E/W	10:1:9	48/52	13 s	B/E/W	10:1:9	48/52	1 m 30 s
	10:2:8	49/51	50 s		10:2:8	52/48	19 s		10:2:8	57/43	>4 m
H/I/W	10:1:9	50/50	>4 m	EA/I/W	10:1:9	50/50	14 s	B/I/W	10:1:9	50/50	15 s
	10:2:8	51/49	24 s		10:2:8	54/46	19 s		10:2:8	58/42	24 s
	10:3:7	52/48	20 s		10:3:7	62/38	22 s		10:3:7	70/30	37 s
	10:4:6	54/46	17 s		10:4:6	74/26	27 s		10:4:6	96/4	1 m 40 s
H/A/W	10:1:9	50/50	14 s	EA/A/W	10:1:9	50/50	10 s	B/A/W	10:1:9	50/50	36 s
	10:2:8	50/50	11 s		10:2:8	56/44	11 s		10:2:8	60/40	43 s

H—*n*-hexane; M—methanol; W—water; E—ethanol; I—isopropanol; A—acetonitrile; EA—ethyl acetate; B—water-saturated *n*-butanol.

**Table 2:** Fraction weights and calculated concentrations.

**Fraction**	**A**	**B**	**C**	**D**	**E**	**F**	**G**	**H**	**I**
Weight (mg)	173.7	44.8	48.6	76.7	119.2	51.5	65.4	25.7	55.6
Weight ratio (%)	4.4	1.1	1.2	2	3.1	1.3	1.7	0.7	1.4
Calculated conc. (μg/mL)	1.33	0.34	0.37	0.59	0.92	0.4	0.5	0.2	0.43
**Fraction**	**J**	**K**	**L**	**M**	**N**	**O**	**P**	**Q**	**R**
Weight (mg)	100.8	106.7	75.8	243.9	116.6	97.2	33.8	82.4	2387.5
Weight ratio (%)	2.6	2.7	1.9	6.2	3	2.5	0.9	2.1	61.1
Calculated conc. (μg/mL)	0.77	0.82	0.58	1.87	0.9	0.75	0.26	0.63	18.34

## Supplementary Materials

The following are available online at https://www.mdpi.com/1420-3049/25/13/3077/s1, Figure S1: Preparation of simulating solvent systems for the gradient elution, Figure S2: CME ARE-luciferase induction activity, Figure S3: CME and marker compound HPLC chromatograms. Table S1: Volume ratio and settling time of biphasic solvent systems, Table S2: The solvent composition in a simulating solvent system of *n*-hexane–acetonitrile–water, ethyl acetate–acetonitrile–water, and water-saturated *n*-butanol–acetonitrile–water (10:2:8, *v*/*v*/*v*).
