# Supplementary material for: Bioassay-Guided Separation of Centipeda minima Using Comprehensive Linear Gradient Centrifugal Partition Chromatography
Source: Molecules. 2020 Jul 6;25(13):3077. doi: 10.3390/molecules25133077 (PMC7412496; doi:10.3390/molecules25133077)
Supplement: Supplementary file 1 [file molecules-25-03077-s001.pdf]

Bioassay-Guided Separation of Centipeda minima using Comprehensive Linear Gradient Centrifugal Partition Chromatography

Ji Hoon Kim [1], Eun Ju Jung [1], Yun Jung Lee [1], En Mei Gao [1], Ahmed Shah Syed [2] and Chul Young Kim [1,*]

[1] College of Pharmacy and Institute of Pharmaceutical Science and Technology, Hanyang University, Ansan, Gyeonggi-do 15588, Korea; gg890718@gmail.com (J.H.K.); jejs2@naver.com (E.J.J.); sopihya@naver.com (Y.J.L.); rhdmsal@hanyang.ac.kr (E.M.G.)

[2] Department of Pharmacognosy, Faculty of Pharmacy, University of Sindh, Jamshoro 76088, Pakistan; shahahmed454@gmail.com (A.S.S)

*Correspondence: chulykim@hanyang.ac.kr; Tel.: +82-31-400-5809; Fax: +82-31-400-5958

Preparation of simulating solvent systems for gradient elution

Preparation of solvent systems for mimetic gradient elution. The lower layer of *n*-hexane-acetonitrile-water (10:2:8, v/v/v) was used as the stationary phase. And then the upper layer of *n*-hexane-acetonitrile-water, ethyl acetate- acetonitrile-water, and water-saturated *n*-butanol-acetonitrile-water (each 10:2:8, v/v/v) were eluted as gradient mode. So simulating solvent systems were prepared as depicted in **Figure 1S**.

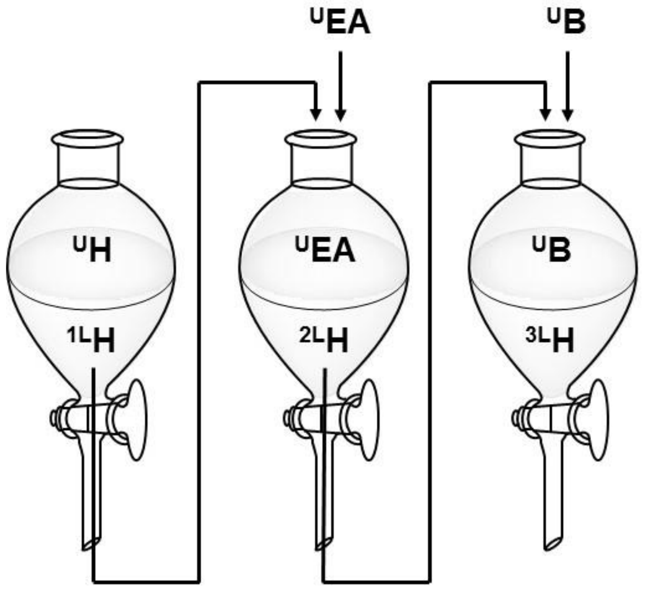

Figure 1S Preparation of simulating solvent systems for the gradient elution. ^{U}H: Upper layer of *n*-hexane-acetonitrile-water (10:2:8, v/v); ^{1L}H: the lower layer of *n*-hexane-acetonitrile-water (10:2:8, v/v/v); ^{U}EA: the upper layer of ethyl acetate- acetonitrile-water (10:2:8, v/v/v); ^{2L}H: the lower layer of mixtures ^{1L}H and ^{U}EA.; ^{U}B: the upper layer of water-saturated *n*-butanol-acetonitrile-water (10:2:8, v/v/v); ^{3L}H: the lower layer mixtures ^{2L}H and ^{U}B.

Table 1S. The volume ratio and settling time of biphasic solvent systems.

Biphasic solvent system		$^{U}H/^{1L}H$	$^{U}EA/^{2L}H$	$^{U}B/^{3L}H$
Volume ratio (%, v/v)	Upper layer	50.0	46.6	47.5
	Lower layer	50.0	53.4	52.5
Settling time (s)		15 s	10 s	35 s

Three kind of ternary biphasic solvent systems were prepared as Figure 1S.

Phase composition analysis of the solvent system by ¹H NMR

The composition of each liquid phase was determined by ¹H-NMR. A 200 μL aliquot of each phase was mixed with 300 μL of $CDCl_3$ for the upper organic phase and $DMSO_{d6}$ for the lower aqueous phase, respectively. Analyses were achieved on a Bruker Avance III 400 spectrophotometer (Bruker, Germany). Spectra were acquired at 298 K with one scan (no dummy scan) over a spectral width of 12 ppm using the standard zq pulse program and a 20 s relaxation delay. The ¹H-NMR signals of *n*-hexane, ethyl acetate, acetonitrile and water, identified by comparison with previously published chemical shifts [1], were integrated in all spectra and the volume percentage (V%) of each solvent was calculated as V%= (A/n) × (MW/d), with **A** the signal area, **n** the number of proton(s), **MW** the molecular weight of the solvent and **d** the density of the solvent at 298 K.

Table 2S. The solvent composition in a simulating solvent system of *n*-hexane-acetonitrile-water, ethyl acetate-acetonitrile-water, and water-saturated *n*-butanol-acetonitrile-water (10:2:8, v/v/v). Simulating solvent systems were prepared described as Figure 1S.

	n-Hexane	Ethyl acetate	*n*-Butanol	Water	Acetonitrile
^{U}H	99.09 %	-	-	0.31%	0.60%
^{1L}H	0	-	-	82.08%	17.92%
^{U}EA	0	74.30%	-	4.90%	20.80%
^{2L}H	0	7.22%	-	83.16%	9.62%
^{U}B	0	6.01%	67.60%	21.10%	5.19%
^{3L}H	0	0.92%	8.90%	85.81%	4.37%

ARE induction activities of CME

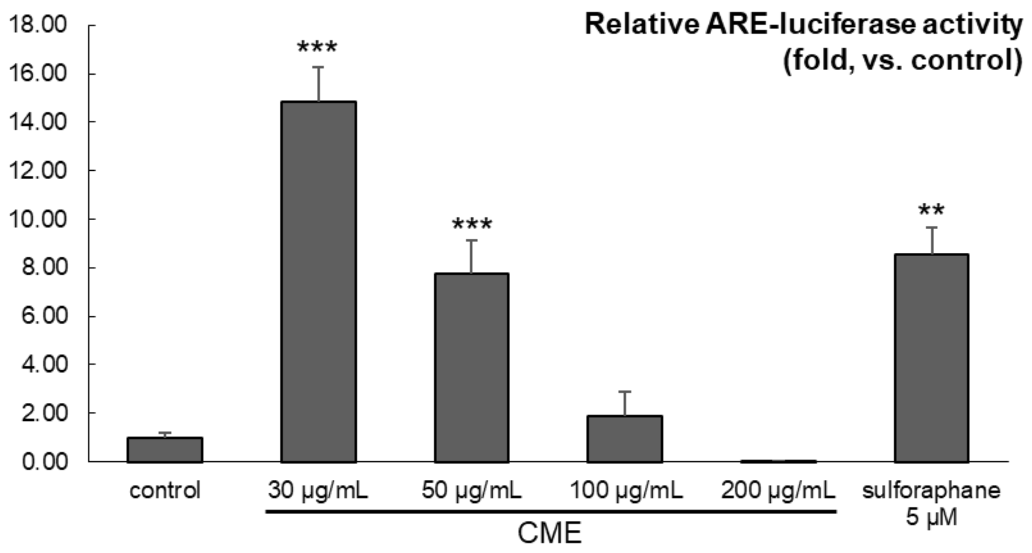

Figure 2S ARE-luciferase induction activity of CME. Relative ARE-luciferase activity of CME serial concentrations (30, 50, 100, 200 μg/ml). Data are presented as means ± S.E. (n = 3). $^{**}P < 0.01$, $^{***}P < 0.005$ were considered statistically significant.

HPLC chromatograms of CME and compounds

In the chromatogram shown in the Figure 3S, the compound **1** and **2** were confirmed based on the information with reference to the literature [1].

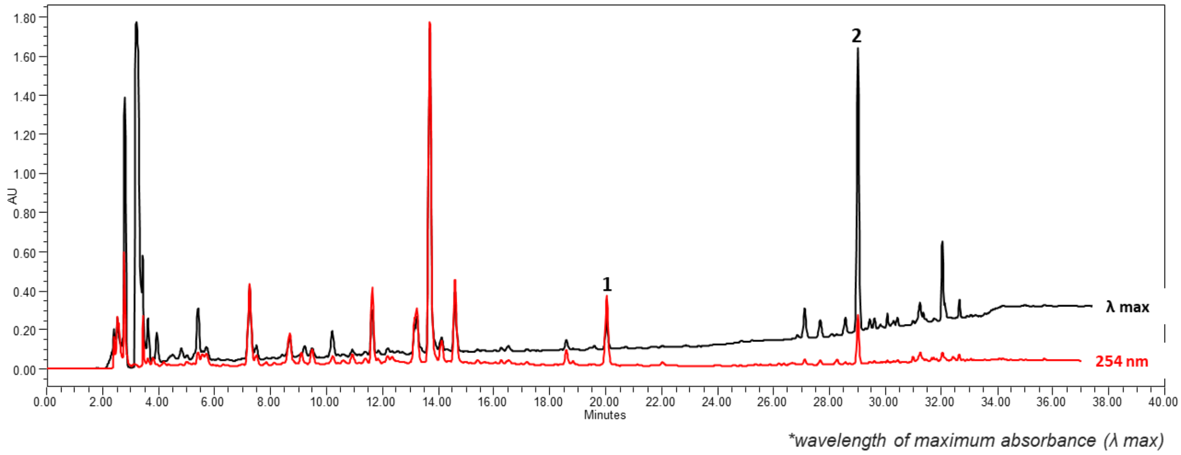

Figure S3 HPLC chromatograms of CME and marker compounds. *Centipeda minima* extract was analyzed by a Waters Alliance 2695 HPLC system coupled with a Capcellpak UG120 C18 column (4.6x250 mm, 5 µm, Shiseido, Japan). Acetonitrile (0.1 % formic acid, solvent A) and water (0.1 % formic acid, solvent B) was eluted in a gradient mode: 0–20 min, 10–40 % A; 20–25 min, 40–55 % A; 25–30 min, 55–95 % A; 40 min, 95 % A. The flow rate was 1 mL/min, and the injection volume was 10 µL. Waters 2996 PDA detector (Waters, Milford, MA, USA) measured the UV spectrum over a range of 200 to 450 nm and the chromatogram of the effluents was recorded at 254 nm and wavelength of maximum absorbance (λ max).

Structural identification

Chemical structures of the purified compound **1** and **2** were determined by ESI-MS and NMR spectroscopic data. The ESI-MS spectra conditions were as follows: positive and negative ion mode; mass range, m/z 100-1200; capillary voltage, 3.5 kV; cone source voltage, 60 V; extractor voltage, 3 V; source temperature, 120℃; desolvation temperature, 350℃; cone gas flow, 50 L/hr; and desolvation gas flow, 900 L/hr. ^{1}H NMR and ^{13}C NMR (400 MHz, or 100 MHz) spectra were measured on a Bruker model digital AVANCE III 400 spectrophotometer (Bruker, Germany). The NMR spectra were processed by the MestReNova 9.0 software (Mestrelab Research, Santiago de Compostela, Spain). The chemical shifts are reported in ppm (δ scale) and all coupling constants (J) values are in hertz (Hz). After comparison of their spectroscopic data with those reported in the literature, the purified compounds were identified as 3-methoxyquercetin (**1**) and brevilin A (**2**). The physicochemical and spectrometric data of two compounds were given as follows.

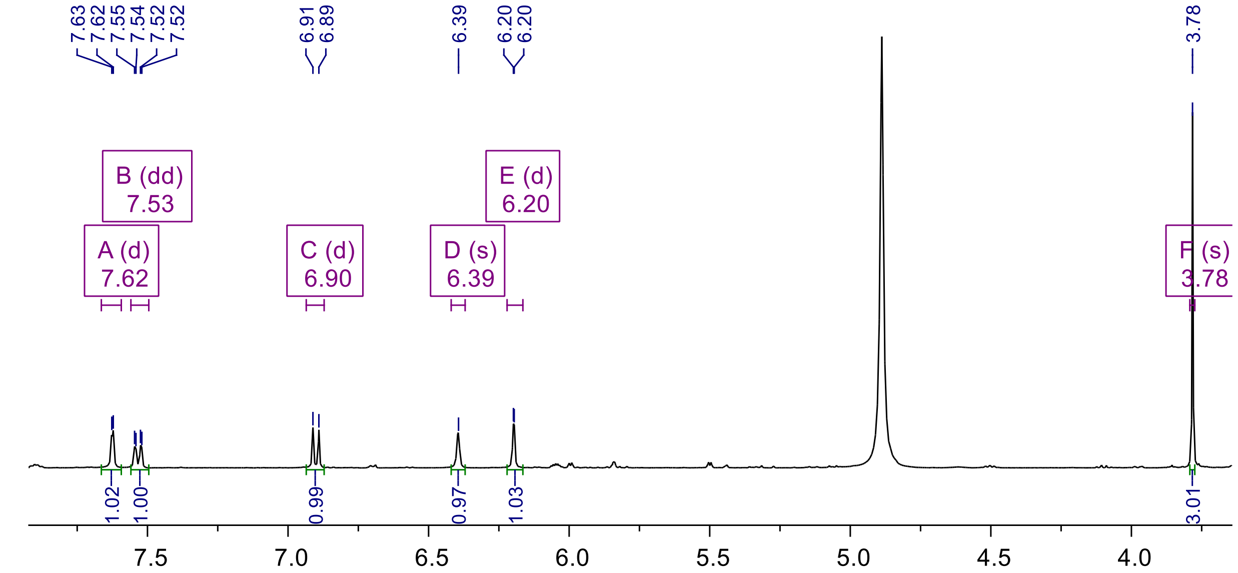

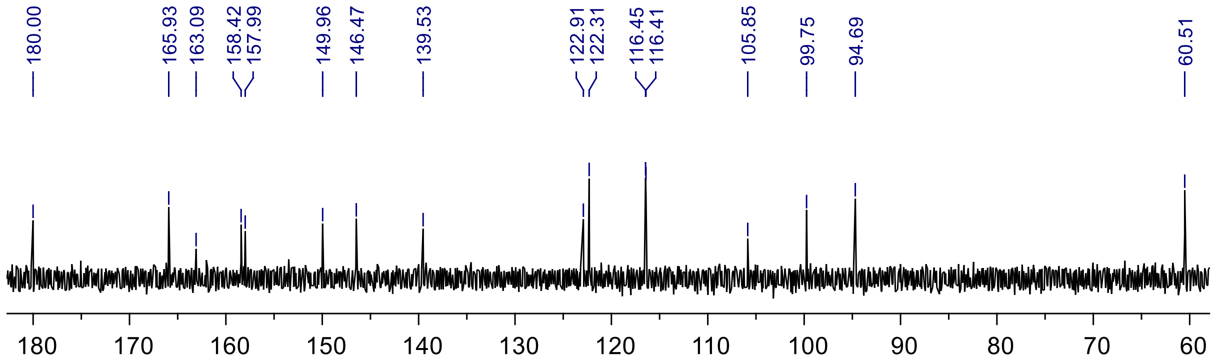

3-methoxyquercetin (**1**). Yellow and needle crystal (MeOH); UV λ-max (MeOH) nm: 255.6, 357.7; ESI-MS m/z 315 $[M-H]^-$; ^{1}H-NMR (MeOD-d_4, 400 MHz) δ (ppm), 3.78 (3H, s, OCH^3-3), 6.20 (1H, d, J = 1.2 Hz, H-6), 6.39 (1H, d, J = 2.1 Hz, H-8), 7.62 (1H, d, J = 2.1 Hz, H-2′), 6.90 (1H, d, J = 8.5 Hz, H-5′), 7.53 (1H, dd, J = 2.0, 8.4 Hz, H-6′); ^{13}C-NMR (MeOD-d_4, 100 MHz) δ (ppm), 158.0 (C-2), 139.5 (C-3), 180.0 (C-4), 165.9 (C-5), 94.7 (C-6), 163.1 (C-7), 99.8 (C-8), 158.4 (C-9), 105.9 (C-10), 122.9 (C-1′), 116.4 (C-2′), 146.5 (C-3′) , 60.5 (OCH^3-3′), 150.0 (C-4′), 116.5 (C-5′), 122.3 (C-6′). The structure was confirmed by comparison with literature data [2].

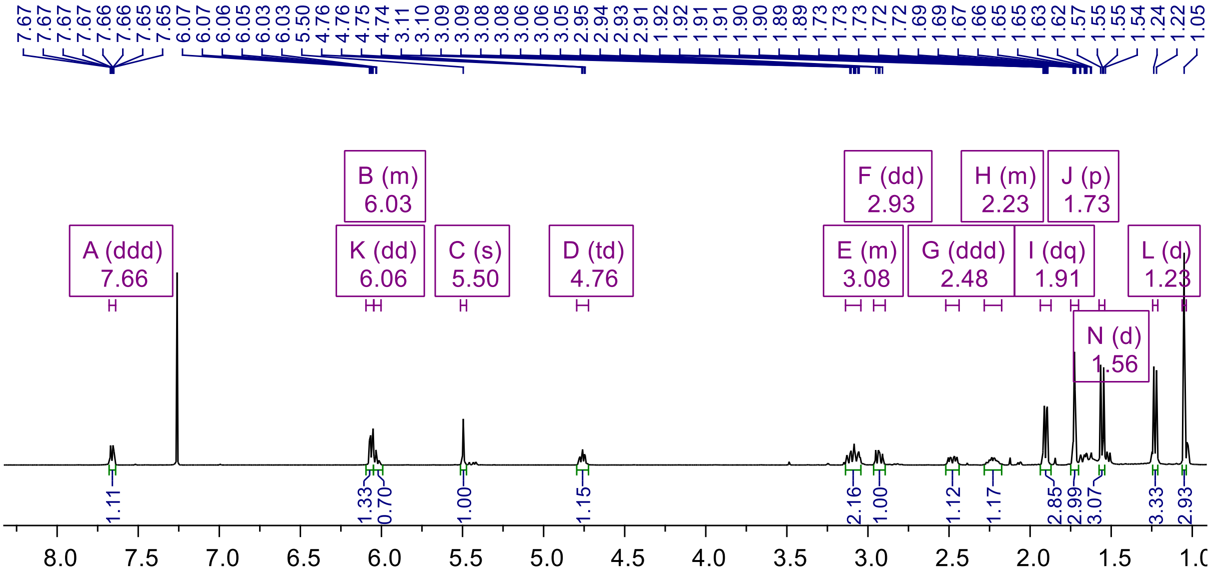

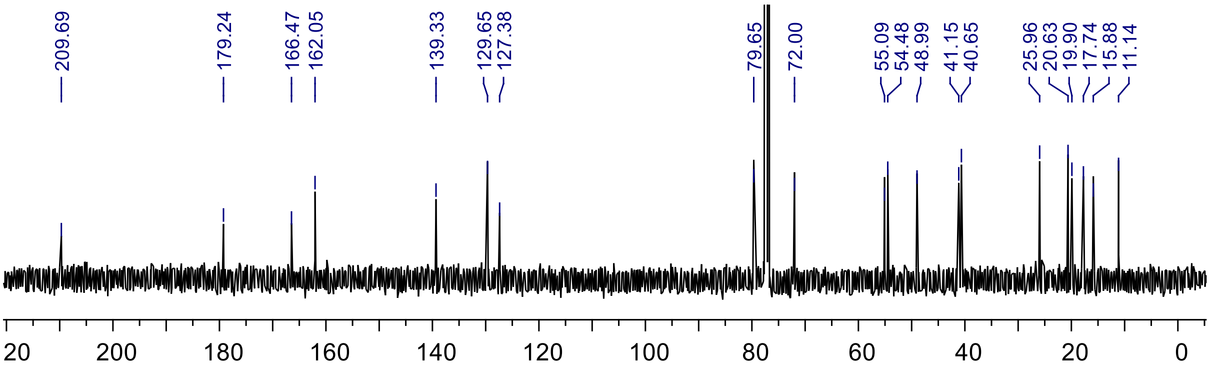

brevilin A (**2**). White powder (MeOH); UV λ-max (MeOH) nm: 223.8; ESI-MS m/z 347 $[M+H]^+$; ^{1}H-NMR ($CDCl_3$, 400 MHz) δ (ppm), 7.65 (1H, dd, *J* = 0.6, 2.0, 6.1 Hz, H-2), 6.06 (1H, dd, *J* = 3.0, 6.0 Hz, H-3), 6.00–6.05 (1H, m, H-3'), 5.48 (1H, s, H-6), 4.75 (1H, td, *J* = 1.9, 6.2 Hz, H-8), 3.04–3.14 (2H, m, H-11, -1), 2.93 (1H, dd, H-7), 2.48 (1H, ddd, *J* = 2.3, 6.0, 15.3 Hz, H-9a), 2.08–2.18 (1H, m, H-10), 1.91 (3H, dq, *J* = 1.5, 7.2 Hz, H-4'), 1.72 (3H, p, H-5'), 1.66 (1H, ddd, *J* = 2.0, 11.1, 15.3 Hz, H-9b), 1.45 (3H, d, *J* = 7.4 Hz, H-13), 1.25 (3H, d, *J* = 6.7 Hz, H-14), 1.01 (3H, s, H-15); ^{13}C-NMR ($CDCl_3$-d_1, 100 MHz) δ (ppm): 54.5 (C-1), 162.0 (C-2), 129.6 (C-3), 209.7 (C-4), 55.1 (C-5), 72.0 (C-6), 49.0 (C-7), 79.6 (C-8), 41.1 (C-9), 25.9 (C-10), 40.6 (C-11), 179.2 (C-12), 11.1 (C-13), 19.9 (C-14), 17.7 (C-15), 166.4 (C-1'), 127.4 (C-2'), 139.3 (C-3'), 15.8 (C-4'), 20.6 (C-5'). The structure was confirmed by comparison with

literature data [1, 3].

References

1. Chan, C. O.; Jin, D. P.; Dong, N. P.; Chen, S. B.; Mok, D. K. W. Qualitative and quantitative analysis of chemical constituents of *Centipeda minima* by HPLC-QTOF-MS & HPLC-DAD. *J Pharm Biomed Anal.* **2016**, 125, 400-407.

2. Wang, J.; Gao, H., Zhao, J.; Wang, Q.; Zhou, L.; Han, J.; Yu, Z.; Yang, F. Preparative separation of phenolic compounds from *Halimodendron halodendron* by high-speed counter-current chromatography. *Molecules* **2010**, 15(9), 5998-6007.

3. On, H. M.; Kwon, B. M.; Baek, N. I.; Kim, S. H.; Lee, J. H.; Eun, J. S.; Yang, J. H.; Kim, D. K. Inhibitory activity of 6-O-angeloylprenolin from *Centipeda minima* on farnesyl protein transferase. *Arch. Pharm. Res.* **2006**, 29(1), 64-66.
